# Identification of single nucleotide polymorphisms in the p21 (CDKN1A) gene and correlations with longevity in the Italian population

**DOI:** 10.18632/aging.100041

**Published:** 2009-04-20

**Authors:** Silvia Gravina, Francesco Lescai, Gregory Hurteau, Graham J. Brock, Anna Saramaki, Stefano Salvioli, Claudio Franceschi, Igor B. Roninson

**Affiliations:** ^1^Cancer Center, Ordway Research Institute, Albany, NY 12211, USA; ^2^Department of Experimental Pathology, Alma Mater Studiorum - Universitá di Bologna, 40126 Bologna, Italy; ^3^CIG - Interdepartmental Centre for Biocomplexity, Alma Mater Studiorum - Universitá di Bologna, 40126 Bologna, Italy; ^4^ITB - Institute for Biomedical Technologies, CNR National Council of Research, 20090 Milan, Italy; ^5^Department of Biochemistry, University of Kuopio FIN-70211 Kuopio, Finland

**Keywords:** CDKN1A, single nucleotide polymorphisms, longevity

## Abstract

Longevity in humans is determined by multiple environmental and genetic factors. We have investigated possible associations between longevity and Single Nucleotide Polymorphisms (SNPs) in the p21 (CDKN1A) gene, a stress-inducible senescence-associated cell cycle inhibitor, expression of which upregulates genes implicated in several age-related diseases. By sequencing the promoter and exons of p21 in genomic DNA of ten individuals over 90 years old, we have identified 30 SNPs, many of which had not been previously characterized. A cluster of minor alleles within the -4547/-3489 bp region did not alter the basal activity or p53 responsiveness of the p21 promoter. We then compared the frequency of 41 p21 SNPs between 184 centenarians and 184 younger subjects in the Italian population. Rare alleles of two exon-derived SNPs, rs1801270 and rs1059234, were significantly under-represented among the centenarians; no significant differences were found for 39 non-exonic SNPs. SNP rs1801270 causes Ser to Arg substitution at amino acid 31 and SNP rs1059234 leads to a nucleotide change in the 3'-untranslated region. Previous studies showed that the rare alleles of these two SNPs may play a role in cancer. These p21 alleles may be potentially detrimental to longevity and therefore are rare in centenarians.

## Introduction

Longevity in humans is believed to be a multifactorial condition to  which  both  genetic and  environmental factors  are likely to contribute.  Centenarians are often spared from age-related diseases, such as cardiovascular disease, Alzheimer's disease, diabetes mellitus, and cancer. The rate of aging and maximum lifespan varies among species, and therefore it has been postulated to be at least in part under genetic control [1-2]. Epidemiological data indicate the presence of a strong familiar component of longevity that is largely determined by genetics. Thus, progeroid syndromes of accelerated aging have known genetic causes [3-4]. A number of possible associations between longevity and allelic variants of genes have been described. Estimates of the heritability of human lifespan vary from 10-50% with the most common finding being that about a third of human lifespan may be heritable. The rest is due to environmental exposure, accidents and injuries, lifestyle and chance. Very long life, to beyond the age of 90 years, appears to have an even stronger genetic basis [5], which explains why centenarians and near-centenarians tend to cluster in families.

Theories on aging postulate that aging is a remodeling process, where the body of survivors progressively adapts to internal and external damaging agents, to which they are exposed over several decades. Thus, stress response and adaptation mechanisms play a fundamental role in the aging process and have an impact on individual lifespan.  Centenarians' capability to live such extraordinarily long lives is in large part due to genetic variations that either affect the rate of aging or decrease the susceptibility to age-associated diseases.

Some of the most promising candidate genes appear to be those involved in stress response. An interesting possible candidate is p21 (CDKN1A) which has been shown to be involved both in stress response mechanisms and in the expression of genes implicated in age-related diseases. p21 is best known as a stress-inducible cyclin-dependent kinase inhibitor, which triggers cell growth arrest associated with senescence and damage response. Some evidence suggests that the effects of p21 inductionon gene expression in senescent cells may contribute to the pathogenesisof cancer and age-relateddiseases. In particular, p21 expression was found to upregulatemultiple genes that have not only been associated with senescence but also implicatedin age-related diseases, including Alzheimer'sdisease, atherosclerosis, amyloidosis, arthritis and cancer, thus suggesting that p21 induction by stress may play a causal role in these diseases [6]. The role of p21 in cell senescence and its possible implication in the risk ofage-related diseases suggests that allelic variations in this gene may have an impact on the lifespan. The goal of this study was to identify p21 polymorphisms and to determine whether they may be associated with longevity.

## Results

### Analysis strategy

To determine if any polymorphic variants of p21 that change either its amino acid sequence or regulation of its transcription may be differentially represented in the long lived individuals (LLI), we carried out this study in several steps. First, in the pilot study, we sequenced the three exons of the p21 gene and a 5-kb stretch of its promoter sequence in the DNA from ten LLI > 90 y.o. (Americans of European descent) to identify SNPs in these regions. To determine if the identified SNPs are specific for LLI, we then used the Sequenom SNP analysis strategy to determine the frequencies of these SNPs in a population of 92 non-LLI individuals (Utah/CEPH population). Finally, in the ethnicity-matched large scale analysis, we used the Sequenom strategy to determine the frequencies of p21 SNPs in ethnically matched Italian populations of 184 LLI and 184 non-LLI control subjects.

### SNPs of the* p21* promoter identified by sequence analysis

As the first approach, we undertook the sequencing of the three p21 exons and a 5-kb sequence upstream of the p21 transcription start site in the genomic DNA from ten LLI. Comparison with the human genome database sequence revealed only one SNP within the three exons, an A->C transversion in codon 31 causing Arg -> Ser substitution (rs1801270). This SNP was previously known and the frequency of the minor allele among the LLI was 0.28 (5/18), which was similar to the minor allele frequency of 0.24 in the general population (unstratified for ethnicity), reported at that time for this SNP in the NCBI database. Promoter sequencing yielded a total of 29 SNPs. Only six of the promoter SNPs had known frequencies reported in the NCBI database, 17 others had been reported but not characterized, and six other promoter SNPs had not been previously reported. To determine the frequencies of the promoter SNPs in a non-LLI population, 25 of these SNPs were assayed in 92 younger Utah/CEPH individuals using Sequenom MassARRAY® system. The positions and allele frequencies for all the SNPs identified in the promoter are presented in Table 1.  Notably, we found ten SNPs that were strongly associated with each other in the Utah/CEPH population  (rs4711458, rs471459, rs4711461, rs4714002, rs471146, rs4714003, rs56850951, rs10947623, rs12192827, rs12192877), in the region between -4547 bp and -3489 bp,  where a novel p53  binding site  has been  recently found [7]. We have found that a cluster of minor alleles within the -4547/-3489 bp region was more common in the ten LLI samples compared to the Utah/CEPH population. The frequencies of the rare allele-carriers (almost all heterozygotes) was 50% among the ten LLIs and 23% for the Utah/CEPH population. This difference did not reach statistical significance (P< 0.158 t-test). No significant differences between these two populations were found for the other SNPs in the promoter region.

**Table 1.** Summary of statistics for SNPs identified in the pilot study.

**SNP**	**Location in the chromosome**	**Rare allele**	**Frequency of rare allele in LLI**	** Frequency of rare allele in controls**	**Common Allele**
*rs4711458*	36749919	C	0.25	0.115	T
*rs4711459*	36750002	C	0.25	0.115	T
*rs4711461*	36750146	T	0.25	0.098	C
*rs4714002*	36750164	T	0.25	0.885	G
*rs471146*	36750168	A	0.25	0.904	G
*rs4714003*	36750238	T	0.25	0.119	C
*rs56850951*	36750380	T	0.25	NA	C
*rs10947623*	36750814	A	0.22	0.118	G
*rs12192827*	36750949	T	0.27	0.120	C
*rs12192877*	36750977	A	0.25	0.080	C
CDKN1A11	36751056	G	0.10	0.070	A
CDKN1A12	36751203	G	0.25	NA	A
CDKN1A13	36751481	A	0.11	0.000	G
rs9394371	36751733	T	0.11	0.110	C
rs4135234	36752199	A	0.12	0.070	G
rs3829963	36752364	A	0.27	0.100	C
rs3829964	36752475	C	0.45	0.400	T
rs3829965	36752488	G	0.27	0.110	A
rs4135237	36752868	T	0.25	0.000	G
rs3829966	36752929	T	0.07	0.070	C
rs3829967	36752936	C	0.07	0.100	T
rs3829968	36752943	C	0.07	0.070	T
rs733590	36753181	C	0.06	0.000	T
rs762623	36753444	A	0.31	0.290	G
rs2395655	36753674	G	0.27	0.320	A
rs730506	36753946	C	0.11	0.190	G
rs4151702	36753966	C	0.11	0.200	G
rs4135239	36754331	C	0.13	NA	G
CDKN1A29	36754348	+C	1.00	NA	

**Figure 1.**
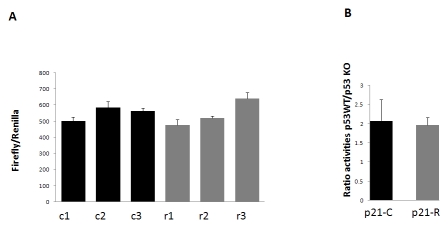
Activities of the p21 promoter-luciferase constructs with the common (p21C-luc; black bars) or rare (p21R-luc; grey bars) allele SNP cluster in the -4547/-3489 bp region.  **(A)** Three independent plasmid preparations of each of p21C-luc or p21R-Luc (R) were transfected into HCT116 wild type (WT) cells. Cells were harvested 48 h after transfection, and firefly luciferase activity was measured and normalized to *Renilla *luciferase expressed from a co-transfected construct. The bars show mean and standard deviation for triplicate transfections. (**B)** p21C-luc and p21R-luc plasmids were transfected in parallel into HCT116 WT and p53-/- cell lines, in triplicates as in A. The bars show mean and standard deviation of the ratio of normalized luciferase activities achieved with the same plasmid in the WT relative to p53-/- cells.

### A cluster of minor alleles in the p21 promoter does not alter its basal activity and p53 responsiveness

Since ten rare SNPs within the region between -4547 bp and -3489 bp, comprising a p53 binding site, were found at a higher frequency among the ten LLI relative to the Utah/CEPH population, we were interested to determine if the presence of these minor alleles affects the basal activity or p53 dependence of the p21 promoter. A 2.1-kb fragment containing a cluster of the minor alleles in this region was amplified by PCR from the genomic DNA of one LLI and cloned into the plasmid p21-PGL.4.10-luc-, which contains 5 kb of the p21 promoter driving the expression of the firefly luciferase reporter gene [7], replacing the common alleles in the corresponding promoter region. The resulting plasmid was designated p21R-Luc. To compare the basal activities of the rare-allele p21R-Luc and the common-allele p21-PGL.4.10-luc construct (designated p21C-Luc in Figure 1), three independent preparations of each plasmid were transfected into wild-type HCT116 colon carcinoma cells, together with a Renilla luciferase expressing vector (normalization standard). Normalized firefly luciferase expression from the two plasmids was indistinguishable, indicating unaltered basal activity of the LLI-derived promoter (Figure 1A). To determine whether the two variants of the p21 promoter could have a different response to p53, we transfected one preparation of each plasmid into wild-type HCT116 cells and into the HCT116 derivative with the knockout of both p53 alleles [8]. The two plasmids showed equal (two-fold) reduction in the promoter activity in p53-knockout cells (Figure 1B), indicating that the LLI-derived promoter had essentially unaltered response to p53.

### Large scale analysis of p21 SNPs correlations with longevity in the Italian population

A comparison of allelic frequencies between the first two population samples that we analyzed, ten LLI from Americans of European origin and younger individuals of the Utah/CEPH population, is inevitably biased by the small sample size of the LLI set and by historically limited variability in the founder pool of the Utah population. Consequently the SNP frequencies could be affected not only by longevity but could also have the founder pool as an uncontrolled confounder. This is an issue typical in genetic association studies affected by the phenomena of ‘stratification': the failure to adequately match the genetic background of cases and controls. Therefore, to minimize this problem, in the large scale analysis we only used DNA from centenarians (mean age 100.88±1.77 years) and younger (38.97±12.21 years) subject populations of Italian origin (184 subjects each), selected for similar origins in Central Italy and representing ethnically matched populations.  We undertook this large case-control design study to (i) verify frequencies of the SNPs identified in the pilot study, (ii) create a haplotype map of 60,000 bp, and (iii) determine whether any specific SNPs and haplotypes are associated with longevity. In addition to 17 of our SNPs identified in the pilot study, 30 SNPs spanning the p21 gene were selected from the SNP HapMap consortium database, for a total of 47 SNPs included in the genotyping.

**Table 2.** Summary of statistics. Large scale analysis of Italian populations. SNPs showing significant differences between the control and LLI populations are shown in boldface; SNPs comprising the sixth haplotype block are italicized. CHISQ=Chi Square, P=P value, OR=Odds

**SNP**	**Location in the chromosome**	**Rare allele**	**Frequency rare allele in LLI**	**Frequency rare allele in controls**	**Common Allele**	CHISQ	**P**	** OR**
rs6457931	36721790	G	0.450	0.447	T	0.006	0.936	1.014
rs1321312	36730852	G	0.155	0.198	C	1.790	0.181	0.746
rs4331968	36731221	T	0.299	0.282	A	0.200	0.655	1.086
rs9470367	36734910	C	0.323	0.321	G	0.004	0.949	1.012
rs6920453	36735461	T	0.228	0.207	C	0.382	0.537	1.134
rs9462209	36736020	G	0.466	0.411	T	1.452	0.228	1.254
rs4713999	36741047	A	0.413	0.459	G	1.098	0.295	0.829
rs1321309	36746614	T	0.440	0.400	C	0.965	0.326	1.177
rs4711459	36750002	C	0.153	0.139	T	0.200	0.655	1.115
rs4711461	36750146	T	0.163	0.136	C	0.826	0.364	1.238
rs4714003	36750238	T	0.086	0.092	C	0.072	0.789	0.921
CDKN1A	36750804	G	0.156	0.181	A	0.667	0.414	0.834
rs10947623	36750814	A	0.165	0.126	G	1.652	0.199	1.366
rs12192827	36750949	T	0.156	0.122	C	1.367	0.242	1.332
rs12192877	36750977	A	0.158	0.134	C	0.679	0.410	1.218
rs4135234	36752199	A	0.151	0.180	G	0.895	0.344	0.809
rs3829963	36752364	A	0.135	0.136	C	0.002	0.969	0.990
rs3829965	36752488	G	0.161	0.142	A	0.378	0.539	1.158
rs4135237	36752868	T	0.158	0.171	G	0.160	0.689	0.913
rs3829966	36752929	T	0.146	0.169	C	0.586	0.444	0.839
rs3829967	36752936	C	0.261	0.212	T	1.974	0.160	1.313
rs733590	36753181	C	0.452	0.458	T	0.025	0.875	0.973
rs762623	36753444	A	0.161	0.188	G	0.705	0.401	0.827
rs2395655	36753674	G	0.479	0.504	A	0.335	0.563	0.904
rs730506	36753946	C	0.245	0.252	G	0.035	0.852	0.964
rs4151702	36753966	C	0.257	0.254	G	0.008	0.930	1.017
*rs3176343*	*36758245*	*A*	*0.044*	*0.075*	*G*	*2.597*	*0.107*	*0.561*
*rs3176344*	*36758525*	*A*	*0.018*	*0.043*	*G*	*3.069*	*0.080*	*0.396*
*rs3176349*	*36759355*	*T*	*0.028*	*0.053*	*G*	*2.091*	*0.148*	*0.526*
***rs1801270***	***36759949***	***A***	***0.043***	***0.093***	***C***	***5.412***	***0.020***	***0.437***
***rs1059234***	***36761575***	***T***	***0.049***	***0.098***	***C***	***4.983***	***0.026***	***0.472***
*rs876581*	*36763423*	*A*	*0.056*	*0.092*	*G*	*2.653*	*0.103*	*0.583*
*rs6457938*	*36768431*	*A*	*0.309*	*0.290*	*G*	*0.255*	*0.614*	*1.096*
*rs6457940*	*36771335*	*A*	*0.284*	*0.311*	*C*	*0.482*	*0.487*	*0.880*
rs2145047	36771630	G	0.037	0.055	A	1.030	0.310	0.661
rs2894409	36774505	T	0.146	0.088	C	4.412	0.036	1.756

**Table 3.** Frequencies and P-values for the sixth p21 haplotype containing SNPs rs1801270 and rs1059234 in the centenarian and control Italian populations.

**Haplotype**	**Frequency LLI**	**Frequency controls**	**P value**	**SNPs**
**Block 6**				
**FOUR SNP WINDOW**				
AGGA	0.03725	0.07798	**0.03972**	rs3176343|rs3176344|rs3176349|rs1801270
GGGA	0.01198	0.01181	0.98520	rs3176343|rs3176344|rs3176349|rs1801270
GGTC	0.02854	0.05105	0.17610	rs3176343|rs3176344|rs3176349|rs1801270
GAGC	0.01811	0.03977	0.12820	rs3176343|rs3176344|rs3176349|rs1801270
GGGC	0.90410	0.81940	**0.00394**	rs3176343|rs3176344|rs3176349|rs1801270
**SIX SNP WINDOW**				
AGGATA	0.03198	0.07607	**0.02249**	rs3176343|rs3176344|rs3176349|rs1801270|rs1059234|rs876581
GGGATA	0.01050	0.01284	0.80000	rs3176343|rs3176344|rs3176349|rs1801270|rs1059234|rs876581
GGTCCG	0.02906	0.05134	0.18560	rs3176343|rs3176344|rs3176349|rs1801270|rs1059234|rs876581
GAGCCG	0.01844	0.03970	0.13900	rs3176343|rs3176344|rs3176349|rs1801270|rs1059234|rs876581
GGGCCG	0.91000	0.82000	**0.00211**	rs3176343|rs3176344|rs3176349|rs1801270|rs1059234|rs876581

**Figure 2.**
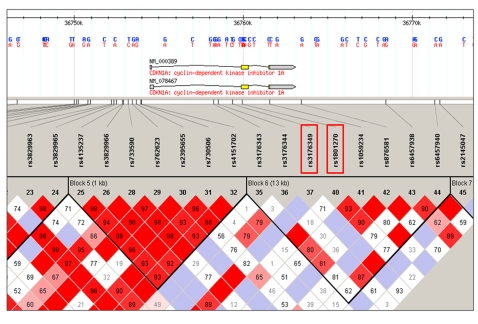
Haplotype blocks distrubution in the p21 gene generated by Haploview. The two SNPs showing significant differences in frequency between the centenarians and younger controls are bracketed. Every multimarker combination within this block including the two SNPs is significant on the omnibus test for frequency distribution among cases and controls. Table 3 gives the results of the haplotype test.

Of those markers, 45 had high confidence calls on the platform and two markers (CDKN1A29 and rs4711458) were excluded because of the low call-rate. Of the remaining SNPs, four (rs4711458, rs4714003, CDKN1A7, rs6920453) were eliminated because the genotype frequencies were not consistent with Hardy-Weinberg equilibrium in the control dataset or in the entire sample. The association statistics for the remaining 41 SNPs are presented in Table 2. Haplotype frequencies were estimated using a sliding window approach. Linkage disequilibrium (LD) analysis of the p21 gene revealed the presence of seven blocks of haplotype in the 60,000 bp region studied. Figure 2 shows a graphical representation of the blocks identified. SNP analysis revealed the presence of two minor alleles that were underrepresented in the LLI compared to the non-LLI control populations, at SNPs rs1801270 and rs1059234. Remarkably, these were the only two exonic SNPs of all the SNPs analyzed. The above mentioned rs1801270 consists of a base change from AGC to AGA and amino acid changes from serine to arginine at codon 31 in exon 2. SNP rs1059234 (p21C70T) consists of a C to T change in the 3' untranslated region of the p21 gene, 20 bp following the stop codon. Minor allele frequencies of SNP rs1801270 were 0.093 and 0.043 in controls and LLI respectively (P< 0.02 chi-test). The corresponding minor allele frequencies for SNP rs1059234 were 0.098 and 0.049 (P< 0.026 chi-test). No statistically significant frequency differences were observed between LLI and controls for the other (non-exonic) SNPs analyzed.

SNPs rs1801270 andrs1059234 are in LD and comprise the sixth LD block in the gene (which includes SNPs rs3176343, rs3176344, rs3176349, rs1801270, rs1059234, rs876581, rs6457938 and rs6457940). Table 3 shows analysis of haplotype structure using tagSNP and estimated haplotype frequencies in this sixth block. A 6-SNP haplotype comprising rs1801270 andrs1059234 common alleles (GGGCCG) is more prevalent in the LLI individuals compared to the controls (91.0% vs 82.0%; p<0.002); the corresponding frequencies for the 4-SNP haplotype of common alleles are 90.4% vs. 81.9% (p<0.004). The significance of these differences was confirmed by permutation analysis.

## Discussion

In the present study, we have investigated possible associations between longevity and SNPs in the p21 (CDKN1A) gene, which plays a role in stress response and cell senescence, and increased expression of which was shown to upregulate genes implicated in several age-related diseases [6]. By sequencing the three exons and 5 kb of the promoter region of p21, we have identified many previously unknown or uncharacterized SNPs in the p21 promoter. We have tested the activity of the promoter derived from an LLI and containing a cluster of minor alleles in the region between -4547 bp and -3489 bp, where a novel p53 binding site has been recently identified [7] and found no changes in the basal activity or p53 responsiveness of this promoter. It should be noted, however, that there are many p53 independent physiological signals that induce p21, where the response of the two alleles may potentially be different. Interestingly the p21 promoter is induced by some signals involved in stress response and inflammation (such as TGFβ, INFγ, IL-6) that, as discussed elsewhere, contribute to the pathogenesis of many age-related diseases [9-11].

In a large case-control design study, we have compared the frequency of 41 SNPs spanning the p21 gene between large populations of LLI and younger Italian individuals. Only two of 41 SNPs showed a statistically significant difference between the two populations, and remarkably, these were the only two exon-derived SNPs. A 6-SNP haplotype comprising the common alleles of these two SNPs was strongly overrepresented among the centenarians relative to the control population. One of these exonic SNPs (rs1801270) changes the amino acid sequence of p21 from Ser to Arg at codon 31, and the other (rs1059234) leads to a C->T transition 20 nucleotides downstream of the stop codon in the 3' untranslated region. Remarkably, several studies sug-gested that the rare alleles of these two SNPs may play a role in different types of cancer [12-17]. In particular, Li et al. [12] have shown that, in non-Hispanic whites, the  rare versions of the rs1801270 and rs1059234 alleles are associated to an increased risk susceptibility to squamous cell carcinoma, individually and in combination. In addition, Mousses at al. [18] observed that the rare alleles of these two SNPs were under-represented in breast cancer and sarcoma patients whose tumors possessed somatic p53 mutations, as compared to tumors without p53 mutations, suggesting that these alleles could influence p21 functions in a p53-independent manner.

According to the data in the NCBI database (Entrez SNP), the allele frequency distribution of the SNPs rs1801270 and rs1059234 is highly variable and ethnic-specific. The frequency of the rs1801270 minor allele varies from 0.021 (Europeans) to 0.47 (Asians) with African-Americans, Sub-Saharan Africans, and Hispanics having intermediate values. Similar values are found for the rs1059234 rare allele, whose frequencies vary from 0.021 (Europeans) to 0.45 (Asians), with African-Americans, Sub-Saharan Africans, and Hispanics also having intermediate values. This high ethnicity-related variability can greatly complicate the interpretation of disease-associated studies, especially those conducted in multi-ethnic societies. In our large-scale study, we have used a relatively homogenous Italian population. Our results suggest that the presence of the rs1801270 and rs1059234 rare alleles combined may be detrimental to longevity and therefore negatively selected in Italian LLI. Further large-scale studies could be useful to compensate for genetic heterogeneity within the Italian population and to clarify the potential role of these SNPs in limiting the lifespan.

The mechanisms underlying the potential detrimental effect of the rare alleles of rs1801270 and rs1059234 are presently unknown. The obvious hypotheses are that the amino acid change at codon 31, which was proposed to abolish p21 phosphorylation at Ser 31 [19], could modulate its abilities to arrest the cell cycle or to induce transcription of genes implicated in age-related diseases, and that a nucleotide change in the 3' UTR could affect p21 mRNA stability or translational efficiency. These possibilities remain to be tested in future studies.

Longevity in humans can be defined as a multifactorial condition to which both genetic and environmental factors are likely to contribute. Twin studies have shown that genetic differences account for about a quarter of the variance in adult human lifespan. Despite the challenges of studying complex traits such as lifespan, studies have been reporting alleles that were significantly associated with human longevity. One of the best examples is APOE whose association has been reproduced consistently [20-22]. The compression of morbidity hypothesis proposed by James Fries in 1980 [23] postulates that as the limit of human lifespan is approached, the onset and duration of lethal impairment compresses toward the end of life. This ‘compression' is observed in the majority of centenarians who are often spared from age-related diseases, specifically cardiovascular disease, Alzheimer disease, diabetes mellitus, and cancer.  To achieve their extreme age, centenarians likely lack numerous gene variants that are associated with age-related diseases and they may be more likely to carry protective variants as well. Our finding that the frequency of specific minor alleles of p21 is decreased among Italian centenarians lends additional support to this concept.

## Materials and methods

Subjects. In the pilot study we used ten DNA samples from LLI over 90 y.o. (white Americans of European descent), including five females and five males. The DNA samples, obtained from the NIA Aging Cell Repository DNA panel, were obtained from CORIELL bank (Camden, NJ). The second group of samples comprised DNA from 92 non-centenarian subjects, belonging to Utah/CEPH population, provided by CORIELL bank. These samples were initially collected from Utah residents with ancestry from northern and western Europe. In the large case-control study, 184 Italians with exceptional longevity (mean age, 100.88±1.77 years) and a control group (38.97±12.21) were recruited by the Bologna group in Central Italy, after checking for ethnicity and ancestor origins.  The sex ratio in the Italian samples was 7 female to 1 male in the centenarian group and 2:1 in the control group. A full socioeconomic, quality of life and health status assessment was performed. Primary criteria for inclusion in the study were good health (for centenarians, subjects categorized A or B according to Franceschi et al. [24] were included), physical activity and absence of major diseases.

p21 genotyping. We sequenced the three exons (68 bp, 450 bp, 1600 bp) and a 5 kb promoter region of the p21 (CDKN1A) gene in the DNA of ten LLI. The exons and the promoter were amplified by PCR in overlapping fragments of ~ 400 bp each. The PCR primer pairs are listed in Table 4, where p21pro.1 is the furthest from the transcription start site. Primers were designed using the* primer3* software available at http://frodo.wi.mit.edu. The primers were sized between 22-24 bases with a Tm of 69-71^o^C and a GC content of 40-60 %. The primers were checked for loops, hairpins and 3' complementarity. The selected primers were synthesized by idtDNA (Coralville, IA). The genomic DNA templates were added to a master mix containing 2 μl of each  primer (10 μM), 5 μl Buffer (New England Biolabs, Ipswich, MA), 2 μl of Taq polymerase (Invitrogen), and water to the volume of 50 μl. The following PCR profile was used: preincubation for 2 min at 96°C, 40 cycles of 30 sec at 95°C, 30 sec at the primer-specific annealing temperature (Table 4) and 15 sec at 72°C, followed by final incubation for 5 min at 72°C. Once amplified, the fragments were purified with Millipore  columns (Millipore Billerica, MA) and then sequenced. Sequencing was performed with ABI 3730 DNA analyzer, using a Big Dye protocol with Zymo column-purified products. Complete sequences were aligned, assembled and compared using the Clone Manager program. For verification, visual inspection of sequence profiles for each candidate SNP was carried out. At least two overlapping DNA templates amplified with different primers were used for identification of each candidate SNP. In addition to the SNP candidate approach, the latest data available on the HapMap were analyzed, in order to select an appropriate number of tagSNPs to cover at least 90% of the genetic information in the locus (r2=0.9 and MAF>0.05). The tagSNP and candidate SNP sets were used for genotyping.

**Table 4.** Primer sequences and TM for amplifying the p21 gene.

**Name**	**Sequence**	**TM**
p21.exon1.R	AAGGCGAGCTCCCAGAAC	60°
p21seq.exon1.F	ACTGGGGGAGGAGGGAAGT	
p21seq.exon2.F	ACCAGCTGGAAGGAGTGAGA	60°
p21seq.exon2.R	GTCTTTGCTGCCTACTTGC	
p21seq.exon3.F1	TGCGGTGATGGATAAAATCA	58°
p21seq.exon3.R1	GAAAAGGAGAACACGGGATG	
p21seq.exon3.F2	TCCTAAGAGTGCTGGGCATT	60°
p21seq.exon3.R2	GCCCTTCTTCTTGTGTGTCC	
p21seq.exon3.F3	TCTTCTCCAGCTGGGCTCT	58°
p21seq.exon3.R3	CCCAAAAGCCCATTTATTTG	
p21pro1.r	GGGGCTGCCTATGTAGTGAA	58°+ dmso
p21pro1.F	GTGCCACAGTTCACAAGTGC	
p21pro2.f	TTTGCTTCTGGGCAGAACTT	58°
p21pro2.r	CAGAGCCAGGATGAATTGGT	
P21.pro.3.f	GATGTTGTTAGAGCCAGGAACAG	54°
P21.pro.3.r	ATCAAGGCATAAAAATTTCATTGTG	
P21pro4f	AAAAGGTTTTTGAATGAATGGATG	58.5°+dmso
P21pro4r.	AGAAGAGGCGGAACAAAGATAGAA	
P21pro5f.	CACGCCCGGCCAGTATATATT TTT	58 °+ dmso
P21pro5r.	GACAAAATAGCCACCAGCCTCTTCT	
p21pro6.f	CACCTTTCACCATTCCCCTA	58°
p21pro6.r	AGGGCTGGTTGTCAAATGTC	
p21pro7.f	TGCATGGTTGCAAACTTTTT	54°
p21pro7.r	TCACCTTTGCCTCCTTTCTG	
p21pro8.f	AGGTCAGCTGCGTTAGAGGA	58°
p21pro8.r	GGAAGGAGGGAATTGGAGAG	
p21pro9.f	GGAGGCAAAAGTCCTGTGTT	54°
p21pro9.r	ACATTTCCCCACGAAGTGAG	
p21pro10.f	TCTAGGTGCTCCAGGTGCTT	58° +dmso
p21pro10.r	CTGTGAACGCAGCACACAC	
p21pro11.f	CCGAAGTCAGTTCCTTGTGG	54°
p21pro11.r	GCTTCCTTGGGAACAAACTG	

The SNP genotyping was performed using Sequenom's chip-based matrix assisted laser desorption/ionization time-of-flight MS (DNA MASSARRAY). This technology performs allele-specific single-base primer extension reactions, which allow differentiation of homozygous normal, heterozygous mutant and homozygous mutant samples (iPLEX assay).  The MassEXTEND primers anneal up to the polymorphic site and are extended with one single base. The allele product masses depend on the SNP allele base and thus they are easily distinguished with the mass spectrometer. DNA of the Utah/CEPH population was analyzed as a service by Sequenom (San Diego, CA) on PCR-derived extension products from individual DNA samples. Large-scale analysis of Italian samples was performed by the Bologna group. Cases and controls were always analyzed on the same chip to avoid potential artifacts caused by chip-specific miscalls.

Plasmid constructs. The plasmid p21-PGL.4.10-luc containing the 5 kb p21 promoter comprising the common alleles and driving firefly luciferase expression has been previously described [7].  This plasmid was used to replace a cluster of common SNP alleles in the promoter with the minor alleles, contained within a fragment of ~2.1 kb, which was amplified by double-round PCR from genomic DNA of a LLI. PCR was carried out using a proofreading polymerase, Phusion™ Hot Start High-Fidelity DNA Polymerase (New England Biolabs, Ipswich, MA). The primed template was pre-formed in the presence of 5X Phusion GC buffer (New England Biolabs, Ipswich, MA) and 200 μM of each dNTP, 0.5 μM primers, and 1 U of Phusion DNA polymerase. The template being GC-rich, 3% DMSO was added to optimize the product yield. The samples were incubated as follows: preincubation for 30 sec at 98°C, 30 cycles of 10 sec at 98°C, 30 sec at 66°C and 3 min at 72°C and one final incubation for 5 min at 72°C. In the first round, the 5kb PCR product of the p21 promoter was amplified using the following primers: p21-4997F   TACAAACATTGGGTGGGGCGAGTC p21-R-44   CTCCGGCTCCACAAGGAACTGACTT In the second round, this PCR product was used as a template to generate a PCR product of ~2.1 kb using the following primers: p21-4497F   TACAAACATTGGGTGGGG CGAGTC p21-5R   GACAAAATAGCCACCAGCCTCTTCT The latter PCR product was digested with AatII and Sph restriction enzymes (New England Biolabs), and cloned into p21-PGL.4.10-luc plasmid digested with the same enzymes, replacing the corresponding fragment containing the common alleles. The resulting plasmid was sequence-verified and designated p21R-luc.

Promoter analysis by transient transfection. HCT116 colon carcinoma cells, both wild type and p53-/- sublines [8] (a gift of Dr. B. Vogelstein, Johns Hopkins University) were grown in DMEM with Earle's salts supplemented with 10% FCS and 2 mM L-glutamine in a humidified 95% air 5% CO2 incubator. Cells were seeded in 12-well tissue culture plates for 24 h prior to transfection. When 70% confluent, the cells were transfected with 1 μg of the indicated promoter-reporter plasmids, together with pRL-TK Renilla luciferase expressing plasmid (Promega, Madison, WI) to normalize for transfection efficiency, at a ratio of 10:1 test vector:standard vector. Transfections were performed in triplicate, using FuGENE6 (Roche Molecular Biochemicals). A precipitate was formed using 3 μl of FuGENE6/μg of transfected DNA and the transfection mixture was diluted up to 1 ml with serum-free medium. After incubation at 22°C for 10 min, the DNA/FuGENE6 mixture was added to cells. Cells were harvested 48 h after transfection, and firefly and Renilla luciferase activities were measured.

Statistical analysis. We tested departurefrom Hardy-Weinberg equilibrium [25] in the controls by a ^2^ testusing *P* = 0.01 as threshold. This threshold was chosen basedon anticonservativeness of this test as noted by Wigginton [25]. All SNPs (except for rs4711458, rs4714003, CDKN1A7, rs6920453) were in Hardy-Weinbergequilibrium. Each SNP was tested both with basic association testing based on comparing allele frequencies between cases and controls (asymptotic and empirical p-value to control for multiple testing), and with Conchran-Armitage trend test in a dominant, recessive and general model.

Multi-locus haplotype analysis was performed by using a sliding-window approach implemented in PLINK [26] for multi-loci of 4 or 6 SNPs size.  Multimarker  haplo- types have been estimated by using the E-M algorithm implemented in the software. An LD map of the region has been produced by using the software Haploview [27] in order to identify LD blocks within the typed markers. The haplotype blocks identified have been tested in the same way, i.e. phased and used for frequency estimation by E-M algorithm and chi-square testing of the frequencies.
